# Effects of Probiotic Mixture Supplementation on the Immune Response to the 13-Valent Pneumococcal Conjugate Vaccine in People Living with HIV

**DOI:** 10.3390/nu13124412

**Published:** 2021-12-09

**Authors:** Marcella Reale, Claudio Ucciferri, Erica Costantini, Marta Di Nicola, Annamaria Porreca, Pamela Di Giovanni, Michela Pontolillo, Antonio Auricchio, Jacopo Vecchiet, Katia Falasca

**Affiliations:** 1 Department of Innovative Technologies in Medicine and Dentistry, University “G. d’Annunzio”, Via dei Vestini, 66100 Chieti, Italy; 2 Department of Medicine and Science of Aging, University “G. d’Annunzio”, Via dei Vestini, 66100 Chieti, Italy; claudio.ucciferri@asl2abruzzo.it (C.U.); erica.costantini@unich.it (E.C.); michy_pontolillo@hotmail.it (M.P.); auricchio.antonio@hotmail.it (A.A.); jacopo.vecchiet@unich.it (J.V.); katia.falasca@unich.it (K.F.); 3 Department of Medical, Oral and Biotechnological Sciences, University “G. D’Annunzio”, Via dei Vestini, 66100 Chieti, Italy; marta.dinicola@unich.it (M.D.N.); annamaria.porreca@unich.it (A.P.); 4 Department of Pharmacy, University “G. d’Annunzio”, Via dei Vestini, 66100 Chieti, Italy; pamela.digiovanni@unich.it

**Keywords:** HIV, immune response, pneumococcal, probiotic, vaccination

## Abstract

Background: In people living with HIV, combination antiretroviral therapy (cART) reduces the risk of death, but the persistent immune-deficient state predisposes them to pneumococcal infections. Current guidelines encourage administering pneumococcal vaccine Prevenar 13 to patients living with HIV. Since probiotic supplementation could act as adjuvants and improve vaccine immunogenicity by modulating gut microbiota, the present study aimed to assess whether the effect of a formulation containing a combination of specific probiotics (Vivomixx^®^) could improve the immune response to 13-valent pneumococcal conjugate vaccine (PCV13) in adult people living with HIV. Methods: Thirty patients who were clinically stable and virologically suppressed, without opportunistic infections during this time and no ART changes in the 12 months before the study started were enrolled. Patients were divided into two groups: (1) received a placebo dose and (2) received Vivomixx^®^ (1800 billion CFU) for four weeks before and after the vaccination with a single dose of PCV13. Results: Vivomixx^®^ supplementation induced a better response to PCV13 immunization, as shown by greater change in anti-Pn CPS13 IgG and increase in salivary IgA, IL-10 and IL-8. Conclusions: Additional investigations will help to clearly and fully elucidate the optimal strains, doses, and timing of administration of probiotics to improve protection upon vaccination in immunocompromised individuals and the elderly.

## 1. Introduction

HIV-infected people continue to have increased morbidity and mortality due to non-AIDS-related events compared to the general population, although combination antiretroviral therapy (cART) reduces the risk of death [1]. HIV-positive (HIV+) patients are a high-risk group for pneumococcal infection due to behavioral and demographic factors. Exposure to infectious agents in the hospital environment, smoking, injection drug use (IDU), alcohol abuse, and immune abnormalities (including the decreased bactericidal activity of neutrophils, reduced phagocytic function of granulocyte and macrophage, CD4+ depletion, altered distribution of T cell subsets, B cell dysfunction, loss of memory B cell subsets, suboptimal humoral immune responses and impaired function of antigen-presenting cells population) increase the risk for invasive pneumococcal disease (IPD) [2,3]. *Streptococcus pneumoniae* is a Gram-positive encapsulated diplococcus that commonly colonizes the upper respiratory tract and can cause mucosal and invasive infections. The highest incidence rates of invasive pneumococcal disease (IPD) are found in young children, older people, and immunocompromised adults [4,5]. Although in HIV+ patients, the immune rebuilding obtained with cART has significantly reduced the incidence of IPD, this rate is still up to 100 times that of the general population [3]. To prevent pneumococcal infection in HIV+ patients, vaccination is recommended, although its efficacy in this patient population has been debated for many years. Vaccination with 23-valent pneumococcal polysaccharide vaccine (PPV23) has provided conflicting results about serological responses and clinical effectiveness. Recently, the pneumococcal conjugate vaccine (PCV) has been added to immunization recommendations for HIV+ subjects [6,7]. The European AIDS Clinical Society (EACS) Guidelines 2021 have indicated the use of one dose of 13-valent pneumococcal conjugate vaccine (PCV13) for all people living with HIV (PLWH) in Europe. PCV13 contains serotypes that are sometimes more prevalent in HIV-infected adults. The conjugation of polysaccharides to CRM197, a non-toxic mutant of diphtheria toxin that acts as a carrier protein, results in a T cell-dependent response. Pneumococcal conjugate vaccines are immunogenic and increase the protection of HIV-infected children against IPD. They have demonstrated efficacy in HIV-infected adults by reducing the risk of pneumococcal disease. Conversely, pneumococcal polysaccharide vaccines have failed to provide these benefits [8,9]. A single dose of PCV13 increased IgG antibody responses as early as one month after vaccination [10]. Accordingly, several countries have given directions for a single dose of the PCV13 vaccine in the vaccination regimen of HIV-infected adults. Studies in mice have confirmed that gut dysbiosis is associated with impaired antibody responses to several vaccines, including the 13-valent pneumococcal conjugate vaccine (Prevenar) [11]. Probiotic administration may correct dysbiosis and enhance innate and adaptive immunities. Even though the specific association between the gut microbiome and vaccine responses is not entirely understood, probiotics may lead to a more efficient protective response [12,13,14]. Probiotic supplementation could act as adjuvants and improve vaccine immunogenicity by modulating gut microbiota. The present study aims to assess if a specific probiotic formulation (Vivomixx) could improve the effects of PCV13 vaccination in HIV+ adults.

## 2. Materials and Methods

### 2.1. Patient Recruitment and Eligibility

Thirty Caucasian male subjects with HIV infection aged 31–66 who were undertaking combined antiretroviral therapy (cART) at the Clinic of Infectious Diseases, Department of Medicine and Science of Aging, “G. d’Annunzio” University (Chieti-Pescara, Italy) were enrolled. Patients were clinically stable and had a constant plasma viral load of <40 copies HIV RNA/mL and a CD4+ cell count of >300 cells/mL during the six months before the start of the study. The patients had not had any opportunistic infections during this time and had been on cART for 12 months before the study started. The patients had not received previous immunization with pneumococcal vaccine in the previous five years. They had not reported episodes of anaphylaxis, hypersensitivity to the pneumonia vaccine, or previous disease/present illness that may affect response to vaccination. Patients had not received blood products or gamma globulin or medications known to affect immune function in the previous three months. Patients excluded from participation were those who were using steroids, growth hormone, testosterone, or any anabolic agent in the previous six months; engaged in drug abuse; or had been treated with other food supplements before the beginning of the study. Subjects were encouraged throughout the study to adhere to their usual diet and lifestyle and report any adverse events or changes in their condition. 

### 2.2. Immunization Protocol and Sample Collection 

**Figure 1:**
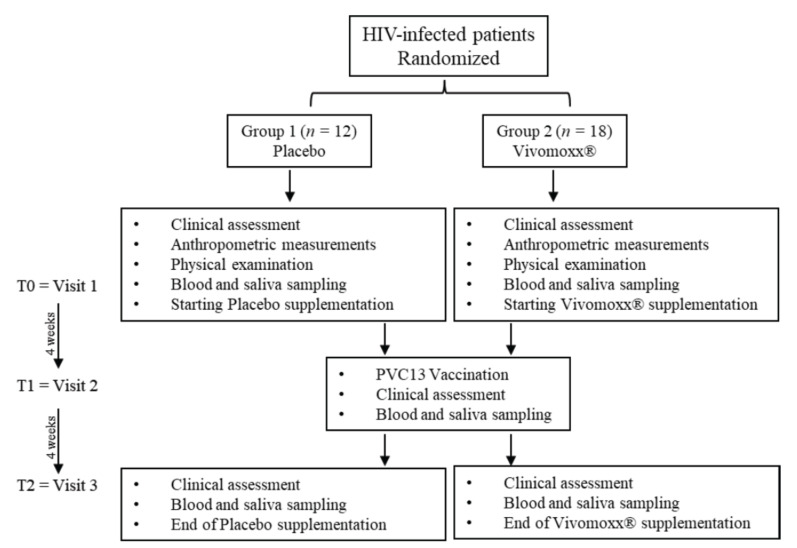
Study design flow chart. Graphical description of randomized sampling.

After agreeing to participate based on written informed consent, a blood sample was taken for biochemical and hematological measurements. Clinical assessment, including anthropometric measurements and physical examination, was performed during the baseline visit. The volunteers were informed that the study would be investigating the effects on immune responses to PCV13 vaccine of a probiotic mix containing *Lactobacillus plantarum* DSM24730(r), *Streptococcus thermophilus* DSM24731(r), *Bifidobacterium breve* DSM24732(r), *L. paracasei* DSM24733(r), *L. delbrueckii subsp. bulgaricus* DSM24734(r), *L. acidophilus* DSM24735(r), *B. longum* DSM24736(r), and *B. infantis* DSM24737(r) available as Vivomixx^®^ (Mendes S.A., Switzerland) in Europe and Visbiome^®^ in USA. Enrolled HIV+ subjects were randomized and divided into two groups: 40% of volunteers received a placebo dose (Group 1), while 60% received two + two sachets a day (each sachet contains 450 billion CFU, total dose per day 1800 billion CFU) of Vivomixx^®^ (Group 2). The product supplementation took place four weeks before the control visit. At the control visit, a single dose of PCV13 vaccine was administered intramuscularly in the deltoid region. There were no cases of pneumococcal disease within one month post-immunization. Both groups were subjected to blood sampling, which involved 10 mL being collected using BD Vacutainer^TM^ tubes with sodium heparin (BD Biosciences, Baltimore, MD, USA) at the basal visit (T0), after four weeks of supplementation and immediately before immunization (T1), and after eight weeks of supplementation and four weeks post-immunization (T2). Serum obtained after centrifugation of peripheral blood was stored at −80 °C until use. Whole unstimulated saliva samples were also collected at each visit. About five mL of unstimulated whole saliva was collected from each subject in sterile polypropylene tubes. The samples were centrifuged at 10,000× *g* for 15 min at 4 °C and the supernatant was immediately aliquoted and frozen (−80 °C), until being analyzed (Figure 1). The study protocol was approved by the Ethics Committee at the University “G. d’Annunzio” Chieti-Pescara (Ethics Committee Project No. 20, 17 October 2019) and was performed under the ethical standards laid down in the 1964 Declaration of Helsinki. 

### 2.3. Biochemical Analyses

Biochemical parameters were measured in blood drawn from patients before starting supplementation and after two months of immunization with PCV-13. Peripheral blood samples were collected into pre-evacuated and light-protected tubes, with no additive or with EDTA, to evaluate levels of high-sensitivity C-reactive protein (hs-CRP); Glycemia (Gly); triglyceride (TG); cholesterol (Chol); high-density lipoprotein (HDL); low-density lipoprotein (LDL); aspartate aminotransferase (AST); alanine aminotransferase (ALT); gamma-glutamyl transferase (GGT); and Creatinemie (CRE).

### 2.4. Virologic and Immunologic Markers

The absolute numbers and proportion (percent) of B cells, T cells, and T cell subsets were determined by flow cytometry (Roche Molecular, Pleasanton, CA). Plasma viral load (HIV-RNA) was determined using the Amplicor method (Roche Molecular, Pleasanton, CA) with a detection limit of >40 HIV RNA copies/mL of plasma.

### 2.5. Human Anti-Pneumococcal CPS13 IgG Measurements

IgG antibodies against capsular polysaccharides (CPS) of 13 serotypes were measured in serum by Human Anti-Pneumococcal CPS13 IgG ELISA Kit (Alpha Diagnostic Intl., San Antonio, TX, USA). These antibodies specifically detect IgG, and do not react with IgM, IgA, or IgE class antibodies. The sensitivity of the assay to detect anti-Pneumococcal (anti-Pn) CPS13 IgG from vaccination is controlled so that the 1 U/mL calibrator represents a threshold O.D. for most true positives in human serum diluted to 1:250. The IgG titers were calculated as the inverse of the dilution that produced a 1.0 O.D. in the assay. 

### 2.6. Salivary IgA Measurements

Secretory IgA concentrations were determined to evaluate the local immunity status in different disease conditions, including respiratory diseases such as pneumococcal disease. The two-site sandwich enzyme immunoassay for secretory IgA was purchased from Bio Vendor Research and Diagnostic Products (Brno, Czech Republic), with a sensitivity assessed as 0.6 µg/mL. The lower detection limit was 57 µg/mL for saliva samples, and the upper limit was fixed at 260 µL/mL.

### 2.7. Cytokines Measurements

Human cytokine levels in serum of HIV+ patients were quantified using specific enzyme-linked immunosorbent (ELISA) assays. According to the manufacturer’s instructions, interleukin (IL-)10 and IL-8 ELISA assays were conducted with commercial kits (Endogen, Woburn, MA, USA). The plates were read at 450 nm, and the absorbances were transformed to pg/mL using calibration curves prepared with cytokine standards included in the kits. The intra- and inter-assay reproducibility was >90.0%. Duplicate values that differed from the mean by greater than 10.0% were not considered for further analysis.

### 2.8. Statistical Analysis

The descriptive statistics for the main characteristics of the study group were expressed as mean and standard deviation (SD) for continuous variables and as absolute frequency (column percentage) for the categorical variables. The normal distribution of data was tested by the Kolmogorov–Smirnov test. The contrasts’ analyses were used to determine mean differences between and within groups, and the *p*-value was reported. This analysis approach was chosen for simplicity over more traditional approaches for analyzing a 2 × 2 factorial design. The Pearson’s chi-squared test was used to compare e proportions between groups. All statistical tests were 2-sided with a significance level set at *p* < 0.05. All analyses were performed with the open-source statistical R environment (version 3.4.3, the R Foundation for Statistical Computing, Vienna, Austria).

## 3. Results

### 3.1. Study Population

The baseline characteristics of participants are outlined in Table 1. All patients infected with HIV-1 were treated with cART according to currently accepted guidelines. Their viral loads were stable and undetectable before the start of Vivomixx^®^ or placebo intake (T0) and throughout the study. All patients well tolerated the addition of Vivomixx^®^ or placebo to their daily diets; no adverse symptoms were reported (fever, nausea or stomach pain, diarrhea). All enrolled patients completed the study. There was 100.0% compliance with the dietary intervention.

### 3.2. Metabolic and Inflammatory Markers

Changes in inflammatory markers and metabolic parameters between groups are shown in Table 2. Our results demonstrate that no significant differences were found in the mean serum concentrations of principal parameters. The principal inflammatory markers hs-CRP did not show any statistically significant differences during the evaluations (Table 2). 

### 3.3. Human Anti-Pneumococcal CPS13

**Figure 2:**
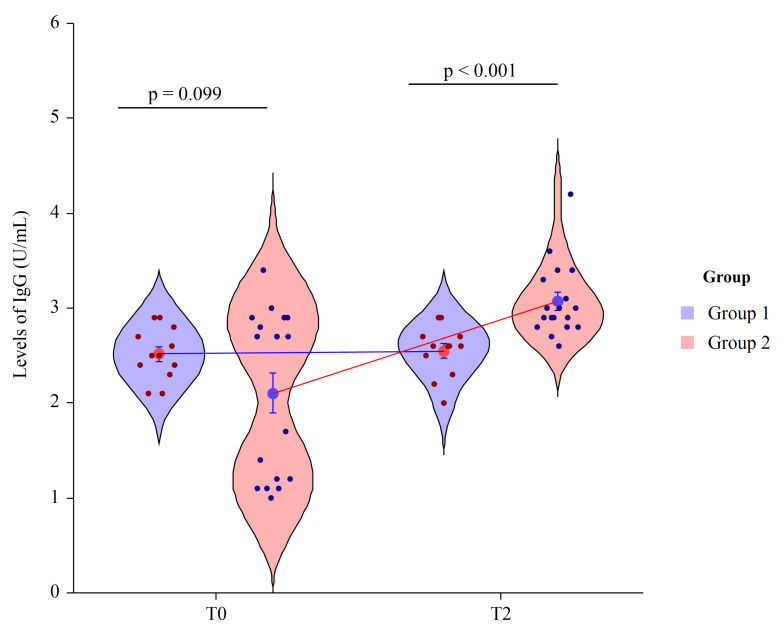
Two factor violin plots for IgG. Data, obtained by ELISA immunoassay, are shown as mean ± SE (standard error) at T0 (basal visit) and T2 (after eight weeks of supplementation and four weeks post-immunization) for both groups of patients. Dots represent the individual data and means are connected by lines. *p*-value derived from planned contrast on *t*-statistic.

We performed an ELISA assay to quantify circulating IgG antibodies against capsular polysaccharides (CPS) of 13 serotypes (Prevenar 13 vaccine) in human serum of vaccinated patients four weeks post-immunization. Since the level of anti-pneumococcus antibodies may increase during opportunistic infections in HIV-1 infected individuals, we considered the evaluation of antibody post-immunization/pre-immunization ratio more accurate than arbitrary levels. Despite a considerable individual variability in responses in all patients, the anti-Pn CPS13 IgG were higher with respect to basal levels. At T0 the mean value of IgG in the Group 1 = 2.5 ± 0.3 and in Group 2 = 2.1 ± 0.9, revealing no statically significant differences (*p* = 0.099). At T2 the mean value of IgG in Group 1 = 2.6 ± 0.3 and in Group 2 = 3.1 ± 0.2, revealing a statically significant difference between groups (*p* < 0.001). Thus, significant immunity was observed from the PCV13 vaccine in patients who had also received the Vivomixx^®^ supplementation with a ratio of 0.9 vs. 0.4 ratio of patients who had received placebo (Figure 2) with a significant variation between groups (*p* < 0.001) at T2.

### 3.4. Salivary IgA

**Figure 3:**
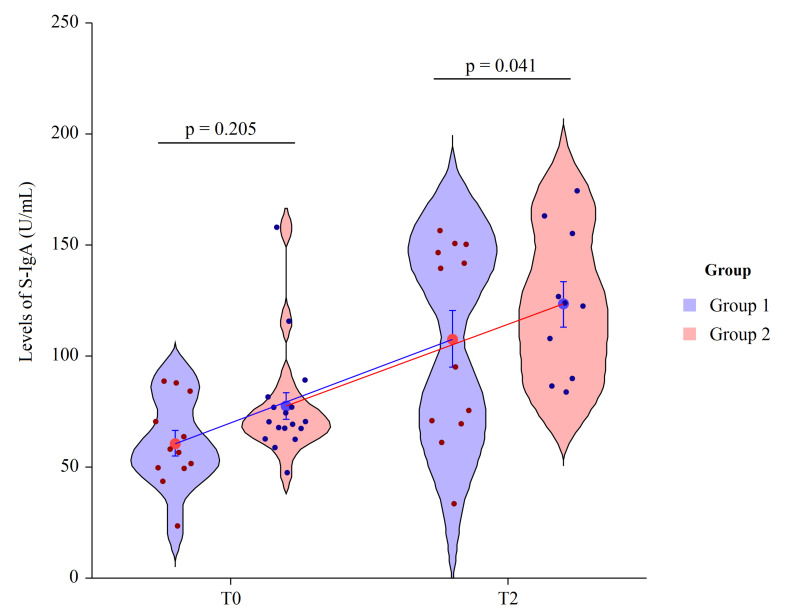
Two factor violin plots for S-IgA. Data, obtained by ELISA immunoassay, are shown as mean ± SE (standard error) at T0 (basal visit) and T2 (after eight weeks of supplementation and four weeks post-immunization) for both groups of patients. Dots represent the individual data and means are connected by lines. *p*-value derived from planned contrast on *t*-statistic.

Secretory immunoglobulin A is the main class of antibodies present in the body’s secretory fluids, such as saliva. Due to its dominance in the immune system of mucus membranes, salivary immunoglobulin A (S-IgA) is typically considered as the first line of defense from environmental factors. Probiotics can increase S-IgA production; we detected an increase of S-IgA in HIV-1 patients supplemented with Vivomixx^®^, with respect to patients not receiving supplements. At T0 the mean value of S-IgA in Group 1 = 60.6 ± 19.6 and in Group 2 = 77.5 ± 25.3, revealing no statically significant differences (*p* = 0.205). At T2 the mean value of S-IgA in Group 1 = 107.6 ± 20.1 and in Group 2 = 123.4 ± 18.5, revealing a statically significant differences in mean value between groups (*p* = 0.041). Thus, after immunization, an additional and different increase was detected. In HIV+ patients, probiotic integration determined an increase of S-IgA ratio of 48-fold (*p* < 0.001) compared to the basal levels, meanwhile in the placebo group, we observed a 35-fold increase (*p* = 0.008) (Figure 3). 

### 3.5. Measures of B Cell Phenotypes 

To determine if probiotic supplementation impacted absolute numbers and percentages of B cells, we tested the peripheral blood after four weeks of placebo or Vivomixx^®^ supplementation immediately before immunization with PCV13. The absolute number of B lymphocyte increased after Vivomixx^®^ integration (214.0 ± 119.0 cells/μL vs. 281.0 ± 209.0 cells/μL) with a 67-fold increase, while in the not supplemented group, B cells number underwent a 12-fold increase. Differences in the percentages of B cells were observed only in Group 2, in which we observed a trend of increase after Vivomixx^®^ supplementation, with significant variation compared to T0 (*p* = 0.004 in T1 vs. T0). For the placebo group (Group 1), a similar value was observed during the time point (Table 3). Furthermore, we evaluated the B cell values and their variation in both groups after four weeks of vaccination. Consistent with the probiotic effect alone, we observed an increase in both groups, with a higher amount of the absolute numbers and percentages of B lymphocytes (*p* < 0.001 in T2 vs. T0) observed in the Vivomixx^®^ supplemented HIV+ patients. 

### 3.6. Measures of Peripheral Blood T Cell Subpopulations 

Four weeks after immunization with PCV13 and eight weeks after beginning supplementation with Vivomixx^®^ (T2), the number of CD4+ and CD8+ cells were not significantly modified. Moreover, in patients who had received placebo, no significant differences were detected at each observation time. In the Vivomixx^®^ supplemented patients, the CD4+ T lymphocytes percentage after immunization (T1) was significantly increased compared to T0, and significantly higher than the CD4+% detected in Group 1 (*p* = 0.002). In addition, four weeks after immunization with PCV13 and eight weeks after beginning supplementation with Vivomixx^®^ (T2), an increase in CD4+% was detected, with significantly higher % than Group 1 (*p* = 0.009). The percentage of CD8+ showed no differences either between times or between groups (Table 4).

### 3.7. Serum Cytokine’s Levels 

The role of cytokines and chemokines as immunomodulatory regulators of neutrophil activity in the presence of pneumococci, IL-10, and IL-8 were evaluated in serum of HIV+ subjects recruited for the study. 

The systemic effect of daily intake of probiotics in HIV+ subjects determined a significant increase of circulating levels of IL-8 with respect to the T0 of the same group (Vivomixx^®^ supplemented) (*p* = 0.009) (Table 5). Moreover, significant differences were observed for Group 2 in relation to Group 1 (placebo) (*p* < 0.001) at T1. However, after four weeks of placebo supplementation, IL-8 levels in Group 1 at the three check times were unmodified. 

Though trending higher, IL-10 serum levels in HIV+ patients supplemented for four weeks with Vivomixx^®^ were not significantly different from basal levels. Instead, we observed a significant increase in IL-10 levels in Group 2 compared to the placebo supplemented subject (*p* < 0.001). After PCV13 vaccination (T2), the serum levels of IL-10 were significantly higher in both Vivomixx^®^ and placebo supplemented patient groups, with a higher increase in Group 2 (*p* < 0.001). Moreover, the increase after Vivomixx^®^ supplementation at T1 was significant also in relation to T0 (*p* < 0.001) (Table 6). 

## 4. Discussion

Immunosuppression is a risk factor for IPD, and low CD4 T cell count is associated with poor response to pneumococcal vaccine. Age may influence immune responses to vaccines [15] since it influences antibody responses’ extent and persistence to pneumococcal capsular polysaccharide [16]. In the elderly, repeated natural exposure to pneumococcal antigens may be associated with hypo-responsiveness to pneumococcus. Moreover, HIV-1 infected subjects present lower vaccination responses and more rapid immunity waning than healthy individuals. Chronic HIV infection causes early immune senescence, and antiretroviral therapy influences the T cell-dependent immunological properties of PCV13 vaccination.

HIV-associated mucosal dysfunction is characterized by impairment of the epithelial barrier, intestinal homeostasis, and microbial translocation other than gut microbiota modifications. Changes in the composition of gut microbiota can be due to modification of species, function, or microbiota–host interactions, contributing to alterations in the intestinal microbiome associated with local and systemic inflammation. In HIV+ subjects, the systemic inflammation is partially related to the CD4+ depletion from the gut-associated lymphoid tissue and the breakdown of microorganism homeostasis in the gut mucosa, with dysregulated immunoregulatory cytokine production [17]. Probiotics can restore composition of gut microbiome ameliorating or preventing gut inflammation and other systemic diseases, through the modulation of immune and inflammatory mechanisms. Recovering the gut microbial population, redirecting the Th2 response, regulating gut Th17 polarization, rising mucus-secretion, improving the IL-10 levels, and maintaining the tight junction proteins regulating lipopolysaccharide (LPS) translocation, are the main functions of probiotics [18]. Thus, in cART-treated HIV+ subjects, probiotic supplementation may reduce microbial translocation, improve gut microbiota, mitigate inflammatory sequelae [19], and improve vaccine effectiveness. 

Different vaccination strategies to improve immunogenicity among HIV-infected adult patients were adopted. Several studies have investigated different response endpoints to pneumococcal vaccination in HIV-infected adults, showing variable immunological outcomes [20,21].

In PCV13, pneumococcal capsular polysaccharides of serotypes 1, 3, 4, 5, 6A, 6B, 7F, 9V, 14, 18C, 19A, 19F, and 23F covalently conjugated to a carrier protein (non-toxic mutant of diphtheria toxin, CRM197) induce systemic immune responses that are highly effective in preventing IPD. The T cell-dependent antibody response to polysaccharide antigens is highly effective in preventing IPD in children and was also recommended for immunocompromised subjects, independently of CD4 count [22]. Although PCV13 covers fewer pneumococcal serotypes than PPV23, it has overcome some, but not all, of the immunological limitations of PPV23 and demonstrated superior immunogenicity in some immunocompromised adults and the elderly [21,23]. However, even though the limited antibody functionality and serotype coverage mean that the benefit of pneumococcal vaccination is less than expected, an attenuated response can also be protective for these individuals. 

Emerging evidence has revealed the gut–lung axis in which gut microbiota may affect pulmonary immunity through vital crosstalk between gut microbiota and the lungs [24]. It has recently been shown that antibiotic-driven dysbiosis in the early life of mice leads to impaired antibody responses to the PCV13 vaccine (Prevenar) [11]. 

Even though the specific mechanisms by which the microbiota affects vaccine responses are not entirely understood, it has been demonstrated that the microbiota constitutes a constant source of natural adjuvants capable of activating many pathways that control innate and adaptive immunity [25]. 

It is known that the most important way by which probiotics may draw health-promoting effects is immunomodulation [19,26]. Thus, this study aimed to evaluate if supplementation with Vivomixx^®^, a well-known and clinically approved commercially available multistrain probiotic, four weeks before and four weeks after vaccination with PCV13 may improve the immunological response in HIV-infected individuals. 

Previous studies on antibody response to PCV13 have highlighted IgG and IgA’s role in the opsonization of pneumococci for phagocytosis [27,28,29]. In this study, we analyzed anti-Pn CPS13 IgG. Because patients with immunodeficiency usually show a widespread response failure across virtually all serotypes, minor differences in a subset of anti-CPS IgG could have little clinical meaning. We considered the evaluation of anti-Pn CPS13 IgG fold differences from baseline more accurate than from arbitrary levels. Our results showed that HIV+ patients had a slight fold change in pneumococcal IgG antibodies in response to PCV13 immunization. However, the Vivomixx^®^ supplementation induced a better response to PCV13 immunization, as shown by greater fold change of anti-Pn CPS13 IgG. 

It is well known that sIgA plays a key role in controlling *Streptococcus pneumoniae* infection in mouse and human studies [30], favoring opsonization and phagocytosis of bacteria. IgA-deficiency reduced pneumococcal vaccine responses and increased recurrence of S. pneumoniae infection. The study on IgA-deficient mice showed colonization by high levels of *Streptococcus pneumoniae* despite the high levels of antigen-specific IgG detected [31].

With regard to salivary IgA antibody, we observed a difference between the groups, with a higher post-vaccination increase in salivary IgA in Vivomixx^®^ supplemented patients, in accord with the study of Lue et al. showing that, of all classes of anti-CPS antibody in saliva and tears, PPV levels increase the most. The authors reported a 2.0-fold increase of salivary IgA levels detected in the saliva of PCV-immunized children [32,33] as result of an immunostimulatory effect of probiotic bacteria [34,35].

Immunological outcomes in our study were comparable with those evidenced by Thitilertdecha et al., which evaluated the impact of immunization on T cells, showing that frequencies and absolute counts of total CD4+ and CD8+ were not significantly different before and after immunization with influenza A vaccine [36]. Moreover, the study of Dell’acqua et al. showed no decrease of CD4+ and CD4+/CD8+ ratio following PCV13 vaccination in antiretroviral-treated people living with HIV [37]. 

In our data, we did not observe any differences in Group 1, while in Group 2, a significant increase in CD4+% values at T1 and a reduction 4 weeks after immunization were detected. Thus, we confirmed that PCV13 immunization did not affect the T cell distribution in antiretroviral-treated people living with HIV. The supplementation with Vivomixx^®^ increased the percentage and absolute values of CD4+ at T1 in Group 2, and four weeks after immunization (T2), a slow reduction was detected, although the percentage and absolute values of CD4+ were higher than values detected in Group 1.

Previous studies have shown that HIV+ patients with high CD4 levels produce a higher concentration of post-vaccination antibodies [38]. These findings are in accord with post-vaccination antibody concentrations detected in our HIV+ subjects supplemented with Vivomixx^®^, who showed an increase in CD4+ count before vaccination and a constant number after vaccination. 

Conjugate vaccines were developed to activate CD4 cells and elicit a T cell-dependent B cell response resulting in memory B cells. To determine if PCV13 immunization impacted B cells, we tested the peripheral blood B cell numbers immediately prior to and four weeks after immunization with PCV13 in Vivomixx^®^ and placebo groups. Our results showed that four weeks of supplementation with Vivomixx^®^ may increase the absolute number and percentage of B cells, and four weeks post-immunization with PCV13, the B cells were higher than in HIV+ patients that received placebo before immunization with PCV13. 

Previous studies have shown that some lactobacilli increase the expression of activation markers on CD8 cells [39]. In our HIV+ patients, we evaluated the CD8^+^ T cells after four weeks of supplementation with Vivomixx^®^. We found that, although CD8^+^ T cells increased following probiotic supplementation, no changes in CD8 T cells were detected after PCV13 vaccination. This is not surprising. It is well known that the main pneumococcal conjugate vaccine mechanism is the induction of CD4+ cells that improve B cells, affinity maturation, class switching, and levels of IgG, while the role of CD8+T cells in resistance to pneumococcus has not been extensively investigated.

During the immune response against *Streptococcus pneumoniae*, the release of several cytokines and chemokines plays a vital role in recruiting neutrophils, monocytes, and lymphocytes to the lungs. In murine pneumonia caused by *Streptococcus pneumoniae* or *Klebsiella pneumoniae*, the anti-inflammatory cytokine IL-10 ameliorated the bacterial clearance from the lung [40]. IL-10, identified initially from Th2 cells, was also expressed in macrophages, mast cells, natural killer cells, neutrophils, and B cells in response to pneumococcal antigens, and shows several immune regulatory effects such as proliferation and differentiation of human B cells, induction of IgA and IgG production by B cells and inhibition of cell-mediated inflammatory reactions. IL-10 during *Streptococcus pneumoniae* infection was produced to modulate the inflammatory response and the immune homeostasis. Gonzales et al. suggest that IL-10 production by neutrophils induced by *Streptococcus pneumoniae* limits lung injury and is essential to mediate an immune response required for host survival [41]. In our HIV+ patients, a weak and non-significant increase of IL-10 was detected after four weeks of diet implementation with Vivomixx^®^. After PCV13 vaccination, a significantly increased IL-10 was detected in both Vivomixx^®^ or placebo supplemented patients, with a higher increase in Vivomixx^®^ group.

Our study shows that after PCV13 vaccination, a chemokine that drives the recruitment of neutrophils to sites of infection was increased, with the exception of IL-10 and IL-8. This supports the results of Madsen et al. postulating the release of IL-8 in response to pneumococcal components [42]. Several preclinical studies have reported that Vivomixx^®^ may modulate the immune response towards an immunoregulatory phenotype. Increased levels of IL-10 and reduced levels of proinflammatory cytokine IL-8 accelerate the normalization of gut immune cells and function in HIV+ patients. Thus, we suggest that the observed effect of Vivomixx^®^ supplementation was probably due to a normalizing effect of the immune system in HIV+ patients.

This preliminary study has some limitations, including a small sample size that could contribute to a lack of statistical significance for some parameters, the non-evaluation of B cell subpopulations, and the antibodies specific to the pneumococcal polysaccharide serotype. Moreover, a limitation is presented by the involvement of only male subjects. It is widely reported that females exhibit more elevated humoral and cell-mediated immune responses to antigenic stimulation, vaccination, and infection than males [43], but data from the Centers for Disease Control and Prevention (CDC) shows that, in 2018, 81% of new HIV diagnoses in the United States and dependent areas were men [44], with a median age of 40 years for both genders.

In accord with these data, 85% of the patients followed at the Clinic of the Infectious Diseases, Department of Medicine and Science of Aging, “G. d’Annunzio” University (Chieti-Pescara, Italy) are males with age ranging from 31 to 66 and given consent to enrollment. 

Our results suggest a role of Vivomixx^®^ in the modulation and improvement of immune responses to the PVC-13 vaccine.

Additional investigations will help to clearly and fully elucidate the optimal strains, doses, and timing of administration of probiotics to improve protection upon vaccination in immunocompromised individuals of both sexes and the elderly. 

## Figures and Tables

**Table 1:** Baseline characteristics of patients. *p*-values are shown in italics.

	Group 1	Group 2	*p*-Value
	*n* = 12	*n* = 18	
Age (years) mean ± SD	48.7± 9.1	47.5 ± 9.3	0.735
BMI (kg/m^2^)	27.0 ± 3.8	24.7 ± 2.0	0.117
Risk factor, n (%)			
eterosex	6 (50.0)	11 (61.1)	0.656
omosex	6 (50.0)	7 (38.9)	

SD, standard deviation. BMI, body mass index.

**Table 2:** Inflammatory and metabolic parameters in HIV+ patients at T0 (basal levels), T1 (after four weeks of supplementation), and T2 (after eight weeks of supplementation and four weeks post-immunization). Data are reported as mean ± SD. *p*-values derived from the contrasts’ analyses are shown in italics.

	Group 1	Group 2	*p*-Value
hs-CRP (mg/100 mL) T0	2.4 ± 0.3	2.6 ± 2.8	0.827
T2	0.9 ± 0.7	7.0 ± 19.7	0.204
Gly (mmol/L) T0	86.5 ± 25.4	92.1 ± 41.9	0.652
T2	97.2 ± 33.5	93.8 ± 30.1	0.784
TG (mg/dL) T0	153.0 ± 108.0	131.0 ± 90.8	0.580
T2	120.0 ± 59.9	126.0 ± 78.9	0.829
Chol (mg/dL) T0	198.0 ± 53.2	209.0 ± 38.9	0.561
T2	200.0 ± 37.5	200.0 ± 31.1	0.970
HDL (mg/dL) T0	49.2 ± 14.4	54.1 ± 10.6	0.335
T2	50.8 ± 15.5	51.1 ± 11.3	0.954
LDL (mg/dL) T0	118.0 ± 37.1	138.0 ± 32.4	0.145
T2	111.0 ± 31.9	129.0 ± 27.1	0.129
AST (IU/L^−1^) T0	34.8 ± 34.5	20.7 ± 7.8	0.190
T2	30.8 ± 25.8	20.4 ± 7.9	0.203
ALT (IU/L^−1^) T0	32.0 ± 15.3	24.8 ± 18.3	0.255
T2	30.9 ± 10.0	21.5 ± 14.2	0.042
GGT (IU/L^−1^) T0	32.5 ± 20.7	26.7 ± 25.6	0.503
T2	34.3 ± 24.0	23.3 ± 16.6	0.182
CRE (mL/min) T0	1.3 ± 1.2	1.0 ± 0.2	0.392
T2	1.5 ± 1.7	1.0 ± 0.2	0.326

hs-CRP, high-sensitivity C-reactive protein; Gly, Glycemia; TG, triglyceride Chol, cholesterol; HDL, high-density lipoprotein; LDL, low-density lipoprotein; AST, aspartate aminotransferase; ALT, alanine aminotransferase; GGT, gamma-glutamyl transferase; CRE, Creatinemie.

**Table 3:** Absolute and % values of B lymphocyte in HIV+ patients at T0 (basal levels), T1 (after 4 weeks of supplementation), and T2 (after 8 weeks of supplementation and 4 weeks post-immunization). Data were reported as mean ± SD. *p*-values derived from the contrasts’ analyses are shown in italics.

		T0	T1	T2
B lymphocytes (%) mean ± SD	Group 1	10.6 ± 4.7	11.1 ± 9.5	12.7 ± 9.0
	Group 2	10.4 ± 4.9	15.5 ± 8.6	19.2 ± 9.5
	*p*-value	0.911	0.180	0.071
B lymphocytes (cells/μL) mean ± SD	Group 1	248.0 ± 91.5	260.0 ± 97.5	329.0 ± 165.4
	Group 2	214.0 ± 59.5	281.0 ± 104.5	307.0 ± 65.5
	*p*-value	0.271	0.580	0.668

SD, standard deviation.

**Table 4:** Percentage and absolute values of T lymphocyte subpopulation in HIV+ patients at T0 (basal levels), T1 (after four weeks of supplementation), and T2 (after eight weeks of supplementation and four weeks post-immunization). Data were reported as mean ± SD. *p*-values derived from the contrasts’ analyses are shown in italics. * indicates the statistically significant difference in respect to the basal time. # indicates the statistically significant difference in respect to the previous observation time.

		T0	T1	T2
CD4+ (%) mean ± SD	Group 1	23.4 ± 9.1	24.5 ± 9.6	23.9 ± 8.5
	Group 2	28.9 ± 3.3	36.9 ± 9.3 *	33.6 ± 7.5 ^#^
	*p*-value	0.066	0.002	0.009
CD8+ (%) mean ± SD	Group 1	45.8 ± 11.0	45.4 ± 10.6	46.0 ± 9.4
	Group 2	39.3 ± 10.8	44.9 ± 5.38	43.9 ± 5.0
	*p*-value	0.123	0.885	0.499
CD4+ (cells/μL) mean ± SD	Group 1	600.0 ± 149.5	612.0 ± 297.8	591.0 ± 178.7
	Group 2	691.0 ± 134.5	820.0 ± 263.9	710.0 ± 154.9
	*p*-value	0.103	0.063	0.063
CD8+ (cells/μL) mean ± SD	Group 1	1073.0 ± 423.0	1093.0 ± 445.0	1097.0 ± 407.0
	Group 2	794.0 ± 266.0	916.0 ± 317.0	872.0 ± 308.0
	*p*-value	0.081	0.079	0.119

SD, standard deviation.

**Table 5:** IL-8 modulation in HIV+ patients at T0 (basal levels); T1 (after 4 weeks of supplementation), and T2 (after 8 weeks of supplementation and 4 weeks post-immunization). Data are reported as mean ± SD. *p*-values derived from the contrasts’ analyses are shown in italics. * indicates the statistically significant difference with respect to the basal time. # indicates the statistically significant difference with respect to the previous observation time.

IL-8	T0	T1	T2
Group 1	47.3 ± 18.3	46.7 ± 15.3	42.4 ± 14.1
Group 2	54.4 ± 25.2	117.0 ± 20.0 *	152.0 ± 15.6 *^#^
*p*-value	0.380	<0.001	<0.001

After receiving PCV-13 immunization (T2), we found a significant difference between the IL-8 levels in serum from the group supplemented with Vivomixx^®^ and the placebo group (*p* < 0.001). However, there were no variations in T2 with respect to T1 for either study group.

**Table 6:** IL-10 modulation in HIV+ patients at T0 (basal levels); T1 (after four weeks of supplementation), and T2 (after eight weeks of supplementation and four weeks post-immunization). Data are reported as mean ± SD. *p*-values derived from the contrasts’ analyses are shown in italics. * indicates the statistically significant difference with respect to the basal time. # indicates the statistically significant difference respect to the previous observation time.

IL-10	T0	T1	T2
Group 1	14.0 ± 25.7	15.3 ± 5.1	18.6 ± 14.0
Group 2	24.1 ± 28.8	96.2 ± 41.1 *	143.0 ± 59.3 *^#^
*p*-value	0.194	<0.001	<0.001

## Data Availability

The data presented in this study are available on request from the corresponding author.

## References

[1] Mazzotta E., Riccardi N., Tontodonati M., Gabrielli C., Mazzocato S., Mazzetti M., Falasca K., Vecchiet J., Barchiesi F., Francisi D. (2019). Prevalence and predictors of malignancies in HIV patients: Results of a retrospective multicentric Italian cohort. Infez. Med..

[2] Titanji K., De Milito A., Cagigi A., Thorstensson R., Grützmeier S., Atlas A., Hejdeman B., Kroon F.P., Lopalco L., Nilsson A. (2006). Loss of memory B cells impairs maintenance of long-term serologic memory during HIV-1 infection. Blood.

[3] Yin Z., Rice B.D., Waight P., Miller E., George R., Brown A.E., Smith R.D., Slack M., Delpech V.C. (2012). Invasive pneumococcal disease among HIV-positive individuals, 2000–2009. AIDS.

[4] Weiser J.N., Ferreira D.M., Paton J.C. (2018). Streptococcus pneumoniae: Transmission, colonization and invasion. Nat. Rev. Microbiol..

[5] Kohli R., Lo Y., Homel P., Flanigan T.P., Gardner L.I., Howard A.A., Rompalo A.M., Moskaleva G., Schuman P., Schoenbaum E.E. (2006). Bacterial pneumonia, HIV therapy, and disease progression among HIV-infected women in the HIV epidemiologic research (HER) study. Clin. Infect. Dis..

[6] Konkle-Parker D. (2014). Vaccination of immunocompromised individuals: IDSA clinical practice guidelines. HIV Clin..

[7] Papadatou I., Spoulou V. (2016). Pneumococcal Vaccination in High-Risk Individuals: Are We Doing It Right?. Clin. Vaccine Immunol..

[8] French N., Gordon S.B., Mwalukomo T., White S.A., Mwafulirwa G., Longwe H., Mwaiponya M., Zijlstra E.E., Molyneux M.E., Gilks C.F. (2010). A trial of a 7-valent pneumococcal conjugate vaccine in HIV-infected adults. N. Engl. J. Med..

[9] Klugman K.P., Madhi S.A., Huebner R.E., Kohberger R., Mbelle N., Pierce N., Vaccine Trialists Group (2003). A trial of a 9-valent pneumococcal conjugate vaccine in children with and those without HIV infection. N. Engl. J. Med..

[10] Bhorat A.E., Madhi S.A., Laudat F., Sundaraiyer V., Gurtman A., Jansen K.U., Scott D.A., Emini E.A., Gruber W.C., Schmoele-Thoma B. (2015). Immunogenicity and safety of the 13-valent pneumococcal conjugate vaccine in HIV-infected individuals naive to pneumococcal vaccination. AIDS.

[11] Lynn M.A., Tumes D.J., Choo J.M., Sribnaia A., Blake S.J., Leong L.E.X., Young G.P., Marshall H.S., Wesselingh S.L., Rogers G.B. (2018). Early-Life Antibiotic-Driven Dysbiosis Leads to Dysregulated Vaccine Immune Responses in Mice. Cell Host Microbe.

[12] Arunachalam K., Gill H.S., Chandra R.K. (2000). Enhancement of natural immune function by dietary consumption of Bifidobacterium lactis (HN019). Eur. J. Clin. Nutr..

[13] Gill H.S., Rutherfurd K.J., Prasad J., Gopal P.K. (2000). Enhancement of natural and acquired immunity by Lactobacillus rhamnosus (HN001), Lactobacillus acidophilus (HN017) and Bifidobacterium lactis (HN019). Br. J. Nutr..

[14] Gill H.S., Rutherfurd K.J., Cross M.L., Gopal P.K. (2001). Enhancement of immunity in the elderly by dietary supplementation with the probiotic Bifidobacterium lactis HN019. Am. J. Clin. Nutr..

[15] Mohr E., Siegrist C.A. (2016). Vaccination in early life: Standing up to the challenges. Curr. Opin. Immunol..

[16] Artz A.S., Ershler W.B., Longo D.L. (2003). Pneumococcal vaccination and revaccination of older adults. Clin. Microbiol. Rev..

[17] Crakes K.R., Jiang G. (2019). Gut Microbiome Alterations during HIV/SIV Infection: Implications for HIV Cure. Front Microbiol..

[18] Cristofori F., Dargenio V.N., Dargenio C., Miniello V.L., Barone M., Francavilla R. (2021). Anti-Inflammatory and Immunomodulatory Effects of Probiotics in Gut Inflammation: A Door to the Body. Front Immunol..

[19] D’Angelo C., Reale M., Costantini E. (2017). Microbiota and Probiotics in Health and HIV Infection. Nutrients.

[20] Ho Y.L., Brandão A.P., de Cunto Brandileone M.C., Lopes M.H. (2013). Immunogenicity and safety of pneumococcal conjugate polysaccharide and free polysaccharide vaccines alone or combined in HIV-infected adults in Brazil. Vaccine.

[21] Lee K.Y., Tsai M.S., Kuo K.C., Tsai J.C., Sun H.Y., Cheng A.C., Chang S.Y., Lee C.H., Hung C.C. (2014). Pneumococcal vaccination among HIV-infected adult patients in the era of combination antiretroviral therapy. Hum. Vaccines Immunother..

[22] Avci F.Y., Li X., Tsuji M., Kasper D.L. (2011). A mechanism for glycoconjugate vaccine activation of the adaptive immune system and its implications for vaccine design. Nat. Med..

[23] Shiramoto M., Hanada R., Juergens C., Shoji Y., Yoshida M., Ballan B., Cooper D., Gruber W.C., Scott D.A., Schmoele-Thoma B. (2015). Immunogenicity and safety of the 13-valent pneumococcal conjugate vaccine compared to the 23-valent pneumococcal polysaccharide vaccine in elderly Japanese adults. Hum. Vaccines Immunother..

[24] Keely S., Talley N.J., Hansbro P.M. (2012). Pulmonary-intestinal crosstalk in mucosal inflammatory disease. Mucosal Immunol..

[25] Pabst O., Hornef M. (2014). Gut microbiota: A natural adjuvant for vaccination. Immunity.

[26] Falasca K., Vecchiet J., Ucciferri C., Di Nicola M., D’Angelo C., Reale M. (2015). Effect of Probiotic Supplement on Cytokine Levels in HIV-Infected Individuals: A Preliminary Study. Nutrients.

[27] Mitra S., Stein G.E., Bhupalam S., Havlichek D.H. (2016). Immunogenicity of 13-Valent Conjugate Pneumococcal Vaccine in Patients 50 Years and Older with End-Stage Renal Disease and on Dialysis. Clin. Vaccine Immunol..

[28] Vandecasteele S.J., De Bacquer D., Caluwe R., Ombelet S., Van Vlem B. (2018). Immunogenicity and safety of the 13-valent Pneumococcal Conjugate vaccine in 23-valent pneumococcal polysaccharide vaccine-naive and pre-immunized patients under treatment with chronic haemodialysis: A longitudinal quasi-experimental phase IV study. Clin. Microbiol. Infect..

[29] Van der Pol W., Vidarsson G., Vilé H.A., van de Winkel J.G., Rodriguez M.E. (2000). Pneumococcal capsular polysaccharide-specific IgA triggers efficient neutrophil effector functions via FcalphaRI (CD89). J. Infect. Dis..

[30] Fukuyama Y., King J.D., Kataoka K., Kobayashi R., Gilbert R.S., Oishi K., Hollingshead S.K., Briles D.E., Fujihashi K. (2010). Secretory-IgA antibodies play an important role in the immunity to Streptococcus pneumoniae. J. Immunol..

[31] Aghamohammadi A., Cheraghi T., Gharagozlou M., Movahedi M., Rezaei N., Yeganeh M., Parvaneh N., Abolhassani H., Pourpak Z., Moin M. (2009). IgA deficiency: Correlation between clinical and immunological phenotypes. J. Clin. Immunol..

[32] Lue C., Tarkowski A., Mestecky J. (1988). Systemic immunization with pneumococcal polysaccharide vaccine induces a predominant IgA2 response of peripheral blood lymphocytes and increases of both serum and secretory anti-pneumococcal antibodies. J. Immunol..

[33] Orami T., Ford R., Kirkham L.A., Thornton R., Corscadden K., Richmond P.C., Pomat W.S., van den Biggelaar A.H.J., Lehmann D. (2020). Neonatal Pneumococcal Conjugate Vaccine Trial team. Pneumococcal conjugate vaccine primes mucosal immune responses to pneumococcal polysaccharide vaccine booster in Papua New Guinean children. Vaccine.

[34] Corthésy B., Gaskins H.R., Mercenier A. (2007). Cross-talk between probiotic bacteria and the host immune system. J. Nutr..

[35] Maidens C., Childs C., Przemska A., Dayel I.B., Yaqoob P. (2013). Modulation of vaccine response by concomitant probiotic administration. Br. J. Clin. Pharmacol..

[36] Thitilertdecha P., Khowawisetsut L., Ammaranond P., Poungpairoj P., Tantithavorn V., Onlamoon N. (2017). Impact of Vaccination on Distribution of T Cell Subsets in Antiretroviral-Treated HIV-Infected Children. Dis. Markers.

[37] Dell’Acqua R., Galli L., Poli A., Mastrangelo A., Guffanti M., Tadini P., Zandona D., Danise A., Gianotti N., Lazzarin A. (2019). Viro-immunological outcomes after 13-valent pneumococcal vaccination in HIV-1-infected individuals on stable virological suppression. AIDS.

[38] Rossheim A.E., Young A.M., Siik J., Cunningham T.D., Troy S.B. (2016). Association of time since pneumococcal polysaccharide vaccine receipt and CD4 count with antibody response to the 13-valent pneumococcal conjugate vaccine in HIV-infected adults. Hum. Vaccines Immunother..

[39] Rask C., Adlerberth I., Berggren A., Ahrén I.L., Wold A.E. (2013). Differential effect on cell-mediated immunity in human volunteers after intake of different lactobacilli. Clin. Exp. Immunol..

[40] Van der Poll T., Marchant A., Keogh C.V., Goldman M., Lowry S.F. (1996). Interleukin-10 impairs host defense in murine pneumococcal pneumonia. J. Infect. Dis..

[41] González L.A., Melo-González F., Sebastián V.P., Vallejos O.P., Noguera L.P., Suazo I.D., Schultz B.M., Manosalva A.H., Peñaloza H.F., Soto J.A. (2021). Characterization of the Anti-Inflammatory Capacity of IL-10-Producing Neutrophils in Response to *Streptococcus pneumonia* Infection. Front Immunol..

[42] Madsen M., Lebenthal Y., Cheng Q., Smith B.L., Hostetter M.K. (2000). A pneumococcal protein that elicits interleukin-8 from pulmonary epithelial cells. J. Infect. Dis..

[43] Fish E.N. (2008). The X-files in immunity: Sex-based differences predispose immune responses. Nat. Rev. Immunol..

[44] Centers for Disease Control and Prevention (2020). HIV Surveillance Report, 2018. http://www.cdc.gov/hiv/library/reports/hiv-surveillance.html.

